# Deep Learning-based Artificial Intelligence Improves Accuracy of Error-prone Lung Nodules

**DOI:** 10.7150/ijms.69400

**Published:** 2022-03-06

**Authors:** Chou-Chin Lan, Min-Shiau Hsieh, Jong-Kai Hsiao, Chih-Wei Wu, Hao-Hsiang Yang, Yi Chen, Po-Chun Hsieh, I-Shiang Tzeng, Yao-Kuang Wu

**Affiliations:** 1 Division of Pulmonary Medicine, Taipei Tzu Chi Hospital, Buddhist Tzu Chi Medical Foundation, New Taipei City, Taiwan.; 2 School of Medicine, Tzu Chi University, Hualien, Taiwan.; 3 Division of thoracic surgery, Taipei Tzu Chi Hospital, Buddhist Tzu Chi Medical Foundation, New Taipei City, Taiwan.; 4 Department of Medical Imaging, Taipei TzuChi General Hospital, Buddhist Tzu Chi Medical Foundation, New Taipei City, Taiwan.; 5 Department of ASUS Intelligent Cloud Services (AICS), ASUSTek Computer Inc.; 6 Department of Chinese Medicine, Taipei Tzu Chi Hospital, Buddhist Tzu Chi Medical Foundation.; 7 School of Post-Baccalaureate Chinese Medicine, Tzu Chi University, Hualien, Taiwan.; 8 Department of Research, Taipei Tzu Chi Hospital, Buddhist Tzu Chi Medical Foundation, New Taipei City, Taiwan.; 9 Department of Research, Taipei Tzu Chi Hospital, Buddhist Tzu Chi Medical Foundation, New Taipei City, Taiwan.

**Keywords:** artificial intelligence, lung nodules, CT images

## Abstract

**Introduction:** Early detection of lung cancer is one way to improve outcomes. Improving the detection of nodules on chest CT scans is important. Previous artificial intelligence (AI) modules show rapid advantages, which improves the performance of detecting lung nodules in some datasets. However, they have a high false-positive (FP) rate. Its effectiveness in clinical practice has not yet been fully proven. We aimed to use AI assistance in CT scans to decrease FP.

**Materials and methods:** CT images of 60 patients were obtained. Five senior doctors who were blinded to these cases participated in this study for the detection of lung nodules. Two doctors performed manual detection and labeling of lung nodules without AI assistance. Another three doctors used AI assistance to detect and label lung nodules before manual interpretation. The AI program is based on a deep learning framework.

**Results:** In total, 266 nodules were identified. For doctors without AI assistance, the FP was 0.617-0.650/scan and the sensitivity was 59.2-67.0%. For doctors with AI assistance, the FP was 0.067 to 0.2/scan and the sensitivity was 59.2-77.3% This AI-assisted program significantly reduced FP. The error-prone characteristics of lung nodules were central locations, ground-glass appearances, and small sizes. The AI-assisted program improved the detection of error-prone nodules.

**Conclusions:** Detection of lung nodules is important for lung cancer treatment. When facing a large number of CT scans, error-prone nodules are a great challenge for doctors. The AI-assisted program improved the performance of detecting lung nodules, especially for error-prone nodules.

## Introduction

Lung cancer is one of the leading causes of cancer-related deaths worldwide 1. The prognosis of lung cancer depends largely on the stage of the tumor. Surgical treatment is the only curative treatment for patients with lung cancer 2. Patients with lung cancer at an operable stage have higher survival rates than those with metastatic disease. Therefore, early detection of early lung cancer is important 1.

The diagnosis and treatment of early stage lung cancer remains challenging. Chest CT is still the main tool used to diagnose lung cancer 3. Using chest CT scans to identify lung nodules may help physicians find early lung cancer. Many efforts have been made to detect lung nodules on chest CT scans to detect early lung cancer 1. Improving the diagnosis of lung nodules on chest CT scans may help diagnose early lung cancer and improve prognosis. Early detection of lung nodules may help in early detection of early lung cancer, which might improve the prognosis of lung cancer patients, and reduce medical costs. However, manually detecting a large number of CT scans is a great burden, requires attention, and is very time-consuming, making it prone to errors. Moreover, lung nodules are sometimes very difficult to detect, even for experienced doctors.

Artificial intelligence (AI) has rapid advantages and exciting achievements in imaging diagnosis. Therefore, many studies have used AI for the detection of lung nodules. These efforts have attempted to improve the accuracy of the detection of lung nodules 1. AI applications have great potential for improving the diagnosis of lung nodules on CT scans. Many of the programs performed well in detecting lung nodules in certain datasets. However, its effectiveness in clinical practice has not been fully proven 1. The low sensitivity or high false-positive rate limits its practical application in clinical practice 1. Therefore, more research is needed to study clinical AI applications.

Therefore, our current research is aimed at using AI-based computer-aided diagnostic systems to help clinicians detect lung nodules on CT scans.

## Materials and methods

### CT acquisition and reading

CT scan images of 60 cases were obtained for the detection of lung nodules. The chest CT scan was performed using a 64-slice detector, GE LightSpeed, and the thickness of the lung window slice was 2.5 mm. Five senior doctors (all had more than 10 years of experience in reading chest CT scans), including three chest physicians, one chest surgeon, and one radiologist, participated in this study for the detection of lung nodules. All of the doctors were blinded to all these cases. Two doctors (doctors 1 and 2) performed manual detection and labeling of lung nodules without AI assistance as a traditional method. Another three doctors (doctors 3, 4, and 5) received AI assistance to detect and label lung nodules before manual interpretation. The study was approved by the Taipei Tzu Chi Hospital, Buddhist Tzu Chi Medical Foundation Institutional Review Board (Protocol Number: 09-X-007).

### Setting and Notations of AI algorithm



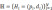





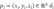





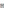





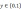



Given a lung 3D CT scan image *I* with* N* nodules, we denote the set of nodules, while 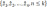 denotes the spatial location and diameter of the *i*th nodule. Weakly supervised pulmonary nodule detection is not accessible during training. Instead, one typically observes the image label from electronic medical records (EMR) during the training stage, which indicates whether the CT scan contains nodules. In our work, we further consider auxiliary information from EMR, including the number *k* of nodules and the slice indices of each nodule on CT scan.

**Figure 1:**
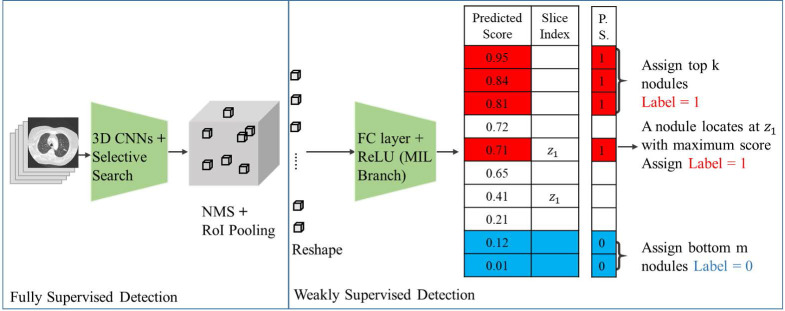
** Framework for pulmonary nodule detection.** The 3D CNN is a pre-trained fully supervised detector that serves as the detector backbone to extract nodule proposals and features in weakly supervised settings. In addition to image-level labels to predict the pseudo labels for each proposal, this model additionally observed nodule numbers and slice index information from EMR to guide the learning process. Abbreviations: 3D CNN: 3-Dimentional convolutional neural network; NMS, non-maximum suppression; RoI pooling, region of interest pooling; FC layer, fully connected layer; ReLU, rectified linear unit; MIL, multiple instance learning; P.S., pseudo labels.

Fig. 1 shows our proposed deep learning framework for weakly supervised pulmonary nodule detection. As shown in Fig. 1, a 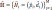 pre-trained nodule of the 3D feature pyramid network (3D-FPN) 1 is applied to extract the preliminary prediction (i.e., features, bounding box location) of each nodule. Such prediction outputs can be viewed as primitive nodule proposals, and the aforementioned weak EMR labels (i.e., image label *y*, nodule number *k*, and nodule slice index *z*) were further utilized to guide the learning of our framework.

### Pulmonary nodule detection with supervision

Previously, multiple instance learning (MIL) 4 has been applied to address object detection in weakly supervised settings, which is realized by observing only image-level labels during training. Without the need to collect any instance-level labels, the above model aims to estimate nodule proposals 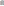, which would be properly associated with the image-level label *y*. For each proposal 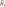, the pooling operation is applied to extract the corresponding feature maps from the 3D-FPN backbone detector, denoted as 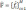. In a previous study 5, fully connected layers with *a rectified linear unit (ReLU)* activation function were deployed to infer the confidence score of each object proposal. Finally, to match the ground-truth image-level prediction, a number of techniques have been proposed to process the predicted 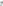 from 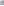 6-8. In our work, we followed and considered the maximum operator as the MIL pooling function:


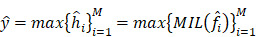

(1)

where *MIL* denotes the MIL branch, and 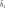 is the predicted score of the proposals in the proposed learning framework. We noted that we fed the extracted visual features into our weakly supervised pulmonary nodule detection module without adjusting the weights of the original ResNet-18 or FPN backbones. This allowed us to focus on the network modules for predicting and re-ranking the extracted nodule proposals under different weak supervision.

The data used for pre-training ResNet 18 comed from the lung nodule open dataset of Lung Image Database Consortium image collection (LIDC-IDRI) (https://wiki.cancerimagingarchive.net/plugins/servlet/mobile?contentId=1966254#content/view/1966254), including 1018 CT volumes from 1010 different patients. A range of scanner manufacturers and models was represented (670 scans from seven different GE Medical Systems LightSpeed scanner models, 74 scans from four different Philips Brilliance scanner models, 205 scans from five different Siemens Definition, Emotion, and Sensation scanner models, and 69 scans from Toshiba Aquilion scanner) 9. After training, we tested the model performance on hospital private data, including 60 CT volumes from 60 patients.

### Reference standard

The lung nodules interpreted by AI and most doctors (at least three doctors) were used as standard references. Sensitivity refers to the rate at which the physician has labeled, and most other physicians and AI are also labeled. Lung nodules labeled by the expert, but beyond the consensus of most other experts, are regarded as false positives (FP) 10.

### Analysis

The overall sensitivity and FP were analyzed. The influence of nodular location (upper, middle, lower; central or peripheral), size, and texture in the CT scan was analyzed.

## Results

### Demographic characteristics

The demographic data of the patients are summarized in Table 1. The mean age was 62.6±11.0 years. The mean body height was 159.8±8.8 cm and the mean body weight was 61.1±13.6 kg. Among them, 27 were males (45%) and 33 were females (55%). Most patients did not smoke (N=44, 73.3%), there were 4 (6.7%) current smokers and 12 (20.0%) former smokers.

### Overall nodular detection

**Figure 2:**
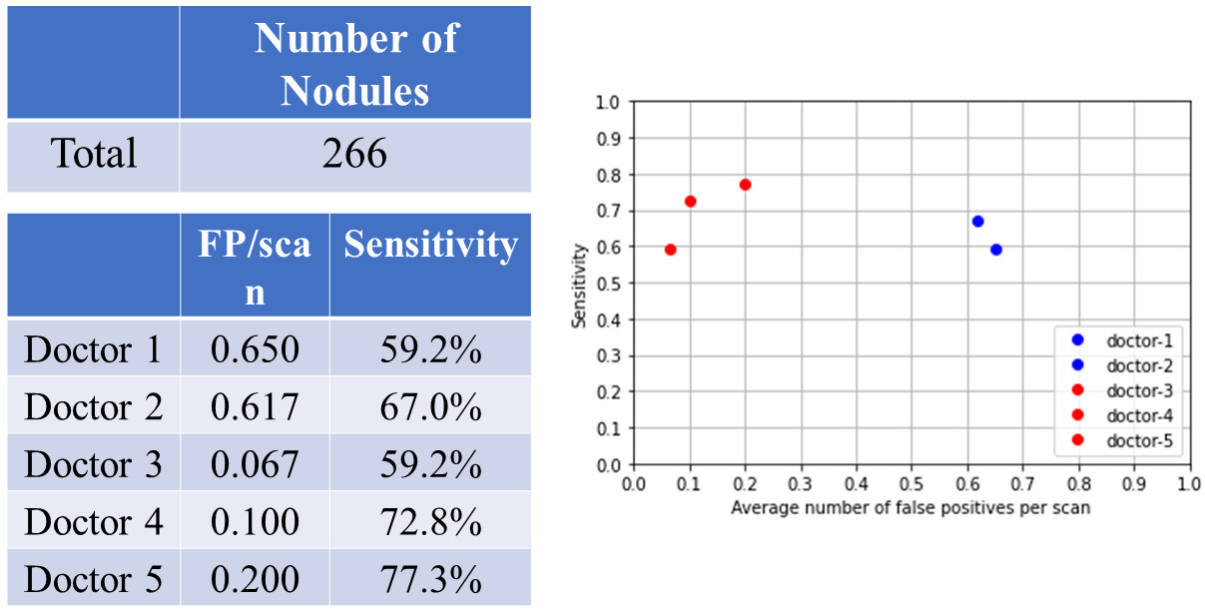
Overall nodular detection.

There were 266 nodules in 60 patients (Fig. 2). For doctors without AI assistance, the FP was 0.617-0.650/scan (mean 0.634, 95% CI 0.586-0.680) and the sensitivity was 59.2-67.0% (mean 63.1%, 95% CI 52.0-74.1%). For doctors with AI assistance, the FP was 0.067 to 0.2/scan (mean 0.122, 95% CI 0.000-0.261), and the sensitivity was 59.2-77.3% (mean 69.8%, 95% CI 50.9-88.6%).

### Left, central and right lung fields and nodular detection

**Figure 3:**
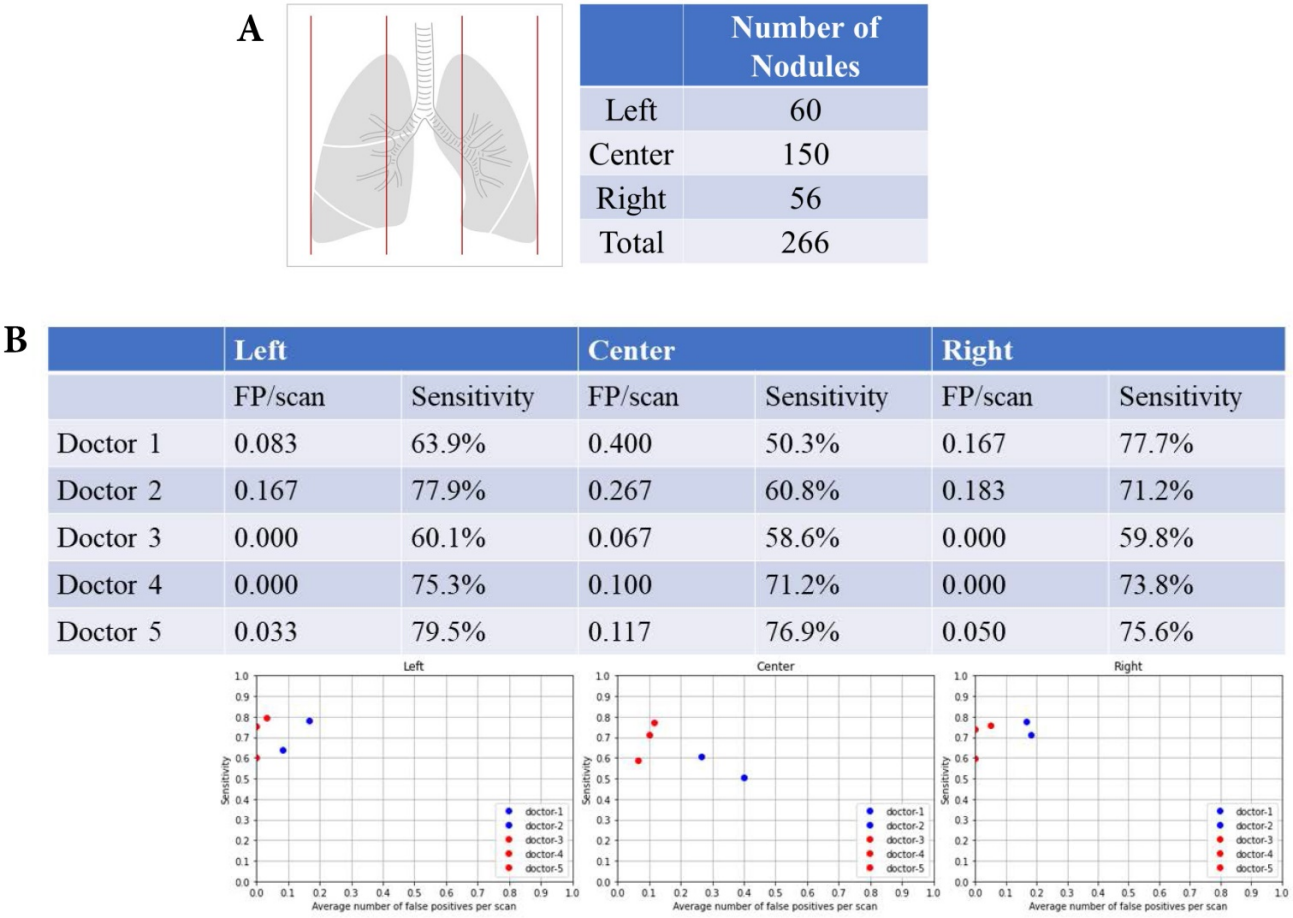
** Left, central and right lung fields and nodular detection.** Number of pulmonary nodular detection in left, center and right lung fields. False positive and sensitivity of nodular detection in left, center and right lung fields.

There were 60 nodules in the left lung fields, 150 nodules in the central lung fields, and 56 nodules in the right lung fields (Fig. 3A). The summary of FP and sensitivity for detecting lung nodules in left, central and right lung fields are shown in Table 2. For the left lung field, the FP was 0.083-0.167/scan (mean 0.125, 95% CI 0.006-0.243) and the sensitivity was 63.9-77.9% (mean 70.9%, 95% CI 51.1-90.6%) without AI assistance (Fig. 3B). With AI assistance, the FP was 0-0.033/scan (mean 0.011, 95% CI 0.000-0.049), and the sensitivity was 60.1-75.9% (mean 71.6%, 95% CI 51.2-92.1%). For the central lung fields, the FP was 0.267-0.40/scan (mean 0.334, 95% CI 0.316-0.352) and the sensitivity was 50.3-60.8% (mean 55.6%, 95% CI 40.7-70.3%) without AI assistance. With AI assistance, the FP was 0.067-0.117/scan (mean 0.094, 95% CI 0.043-0.145) and the sensitivity was 58.6-76.9% (mean 68.9%, 95% CI 50.1-87.6%). For the right lung field, the FP was 0.167-0.183/scan (mean 0.176, 95% CI 0.153-0.197) and the sensitivity was 71.2-77.7% (mean 74.5%, 95% CI 65.2-83.6%) without AI assistance. With AI assistance, the FP was 0-0.05/scan (mean 0.017, 95% CI 0.000-0.074) and the sensitivity was 59.8-75.6% (mean 69.7%, 95% CI 52.4-87.0%).

### Upper, middle and lower lung fields and nodular detection

**Figure 4:**
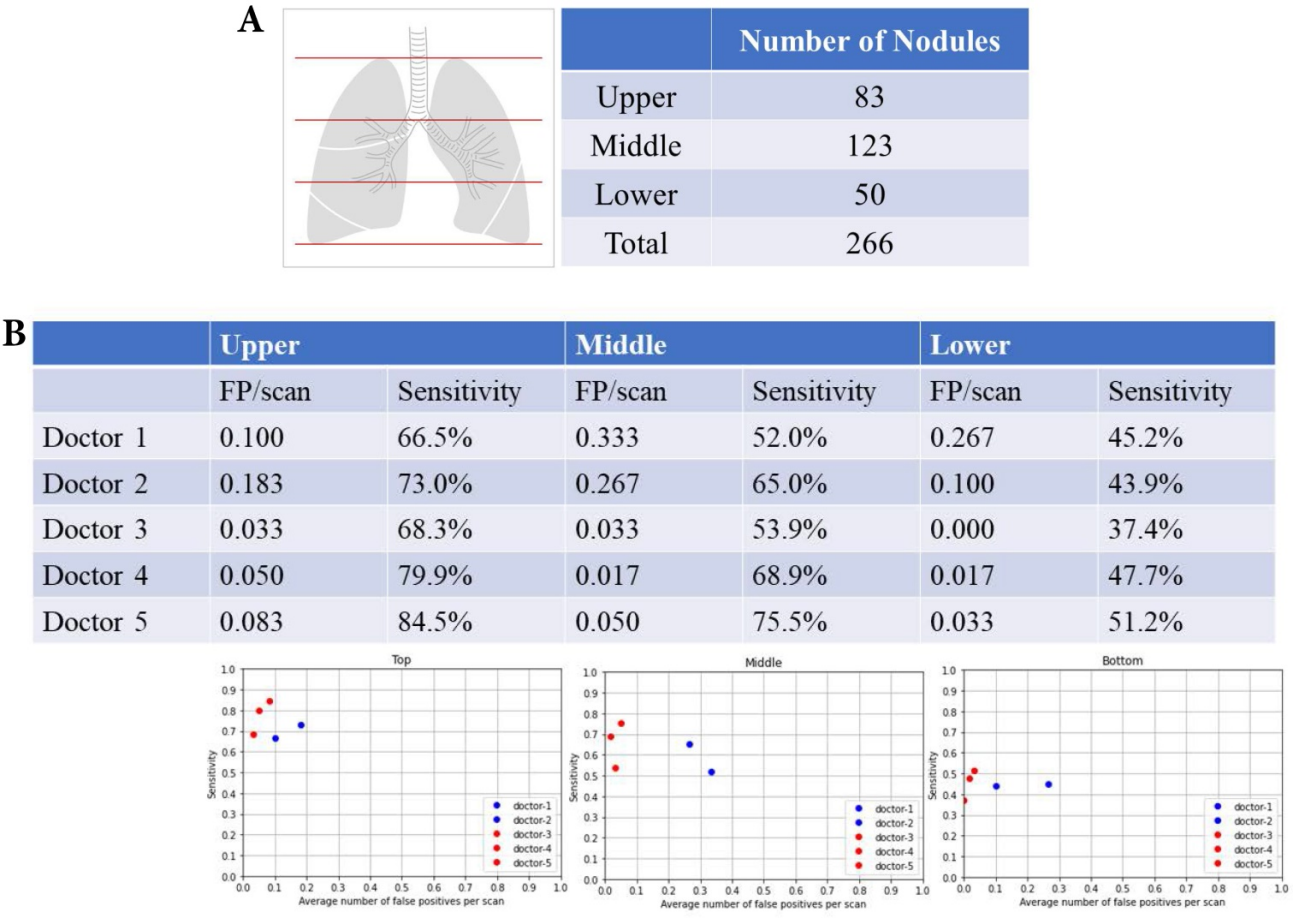
** Upper, middle and lower lung fields and nodular detection. (A)** Number of pulmonary noular detection in upper, milddle and lower lung fields. **(B)** False positive and sensitivity of nodular detection in upper, milddle and lower lung fields.

There were 83 nodules in the upper lung fields, 123 nodules in the middle lung fields, and 50 nodules in the lower lung fields (Fig. 4A). The summary of FP and sensitivity for detecting lung nodules in upper, middle and lower lung fields are shown in Table 2. For the upper lung fields, the FP was 0.100-0.183/scan (mean 0.142, 95% CI 0.024-0.258) and the sensitivity was 66.5-73.0% (mean 69.8%, 95% CI 60.5-78.9%) of doctors without AI assistance and FP was 0.033-0.083/scan (mean 0.055, 95% CI 0.004-0.106), and the sensitivity was 68.3-84.5% (mean 77.6%, 95% CI 60.8-94.2%) for doctors with AI assistance (Fig. 4B). For the middle lung fields, the FP was 0.267-0.333/scan (mean 0.300, 95% CI 0.206-0.393) and the sensitivity was 52.0-65.0% (mean 58.5%, 95% CI 40.1-76.8%) of doctors without AI assistance and FP was 0.017-0.050/scan (mean 0.033, 95% CI 0.003-0.066) and the sensitivity was 53.9-75.5% (mean 66.1%, 95% CI 43.9-88.2%) of doctors with AI assistance. For the lower lung fields, the FP was 0.1-0.267/scan (mean 0.184, 95% CI 0.000-0.419) and the sensitivity was 45.2-43.9% (mean 44.5%, 95% CI 42.7-46.3%) of doctors without AI assistance and FP was 0.017-0.033/scan (mean 0.017, 95% CI 0.000-0.049) and the sensitivity was 37.4-51.2% (mean 45.4%, 95% CI 31.0-59.7%) of doctors with AI assistance.

### Nodular size and nodular detection

**Figure 5:**
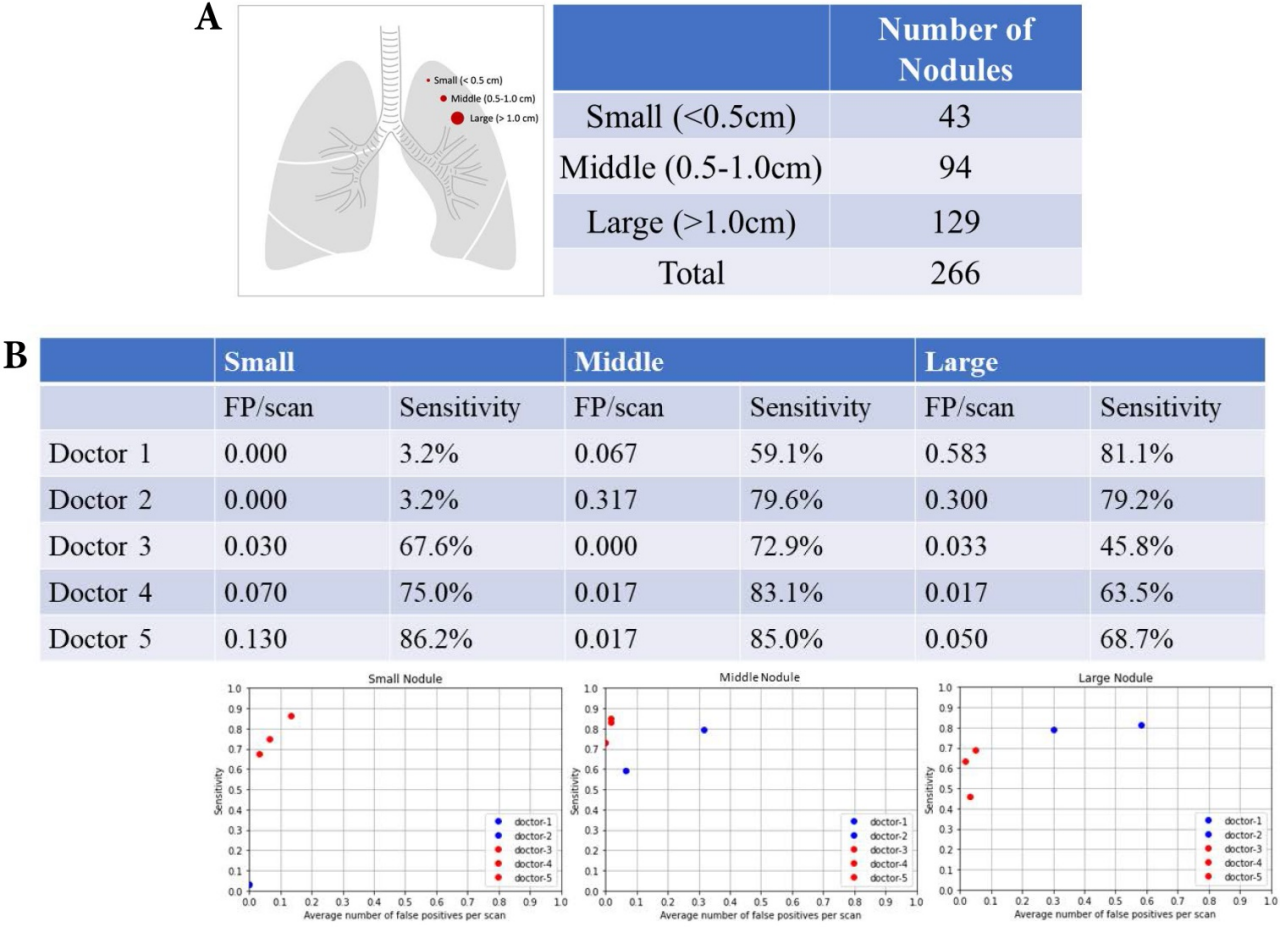
** Nodular size and nodular detection. (A)** Number of difference sizes of pulmonary nodules. **(B)** False positive and sensitivity of nodular detection in difference sizes of pulmonary nodules.

There were 43 small nodules (diameter <0.5 cm), 94 middle nodules (diameter 0.5-1.0 cm), and 129 large nodules (diameter >1.0 cm) (Fig. 5A). The summary of FP and sensitivity for detecting lung nodules of different nodular sizes is presented in Table 2. For the small nodules, the FP was 0 (mean 0, 95% CI 0-0) and the sensitivity was 3.2% (mean 3.2%, 95% CI 3.2-3.2%) of doctors without AI assistance and FP was 0.003-0.13/scan (mean 0.077, 95% CI 0.000-0.177), and the sensitivity was 64.6-86.2% (mean 76.3%, 95% CI 57.5-94.9%) of doctors with AI assistance (Fig. 5B). For the middle nodules, the FP was 0.067-0.317/scan (mean 0.192, 95% CI 0.000-0.545) and the sensitivity was 59.1-79.6% (mean 69.4%, 95% CI 40.3-98.3%) of doctors without AI assistance and FP was 0-0.017/scan (mean 0.011, 95% CI 0.000-0.030), and the sensitivity was 72.9-85.0% (mean 80.3%, 95% CI 67.3-93.3%) for doctors with AI assistance. For the large nodules, the FP was 0.3-0.583/scan (mean 0.442, 95% CI 0.041‒0.841) and the sensitivity was 79.2-81.1% (mean 80.2%, 95% CI 77.4-82.8%) of doctors without AI assistance and FP was 0.033-0.050/scan (mean 0.033, 95% CI 0.000-0.066) and the sensitivity was 45.8-68.7% (mean 59.3%, 95% CI 35.3-83.3%) of doctors with AI assistance.

### Nodular texture and nodular detection

**Figure 6:**
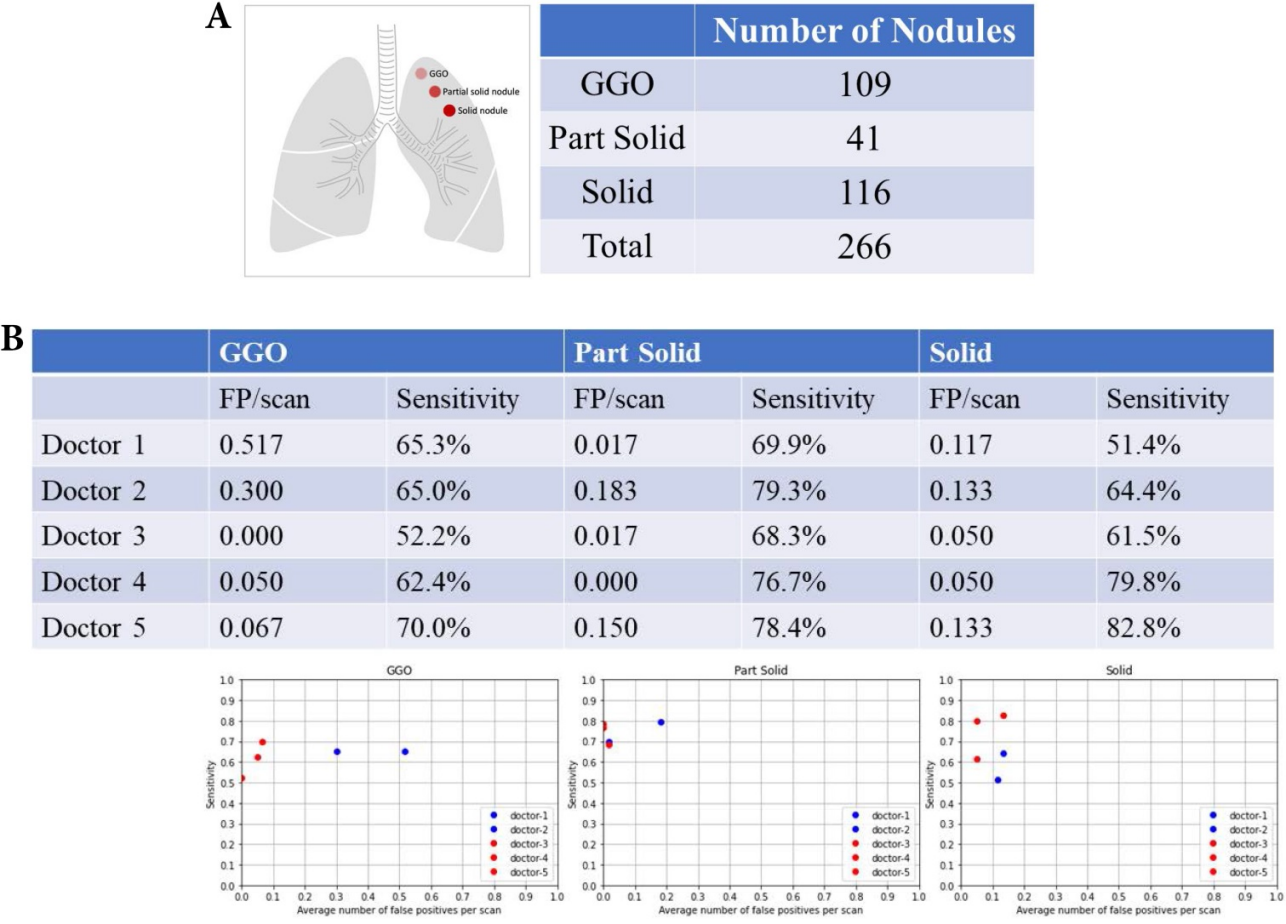
** Nodular texture and nodular detection.** Number of difference textures of pulmonary nodules. False positive and sensitivity of nodular detection in difference textures of pulmonary nodules.

There were 109 ground-glass organization (GGO) nodules, 41 partial nodules, and 116 solid nodules (Fig. 6A). Table 2 summarizes the FP and sensitivity for detecting lung nodules of different nodular textures. For the GGO nodules, the FP was 0.3-0.517/scan (mean 0.409, 95% CI 0.101-0.715) and the sensitivity was 65.0-65.3% (mean 65.1%, 95% CI 64.7-65.5%) of doctors without AI assistance and FP was 0-0.067/scan (mean 0.039, 95% CI 0.000-0.108) and the sensitivity was 52.2-70.0% (mean 61.5%, 95% CI 43.6-79.3%) of doctors with AI assistance (Fig. 6B). For the partial solid nodules, the FP was 0.017-0.183/scan (mean 0.100, 95% CI 0.000-0.334) and the sensitivity was 69.9-79.3% (mean 74.6%, 95% CI 61.3-87.8%) of doctors without AI assistance and FP was 0-0.150 (mean 0.056, 95% CI 0.000-0.219) and the sensitivity was 68.3-78.4% (mean 74.5%, 95% CI 63.6-85.2%) of doctors with AI assistance. For the solid nodules, the FP was 0.117-0.133/scan (mean 0.125, 95% CI 0.102-0.147) and the sensitivity was 51.4-64.4% (mean 57.9%, 95% CI 39.5-76.2%) of doctors without AI assistance and FP was 0.050-0.133/scan (mean 0.078, 95% CI 0.000-0.173), and the sensitivity was 51.2-63.5% (mean 74.7%, 95% CI 51.6-97.7%) of doctors with AI assistance.

## Discussion

The current study has several important findings. Doctors who are not assisted by AI are more likely to have more FP and less sensitivity in their predictions with respect to the center and middle positions. Facing small lung nodules (less than 0.5 cm), the doctor's sensitivity is quite poor. In terms of nodule texture, doctors were more likely to have more FP for GGOs. With the assistance of AI, the overall false positive and sensitivity of the doctor's interpretation can be improved. The accuracy of the above-prone areas or features can also be improved.

A reasonable idea is that if the nodule is too small, the doctors or the AI may easily miss the lung nodules. Previous studies have found that the most common cause of missed diagnosis on CT scans is its small size 11. Del Ciello et al. suggested that a small diameter (<7 mm) is one of the causes of failed diagnosis 11. In our study, the tiny nodules (< 0.5 cm) were quite difficult to manually detect in chest CT scans with a sensitivity of only 3.2%. With AI assistance, the sensitivity was greatly improved for tiny nodules. Several studies have also demonstrated that AI, as a second reader, significantly increases sensitivity in the identification of lung nodules 12.

One of the factors leading to missed lung nodules is their central location 11. Del Ciello et al. reported that the missing rate of lung nodules in the central area is disproportionately high 11. Deveraj et al. also found that hilar nodules, that are blind spots on CT scans, are one of the causes of missed lung cancers 13. Some normal tissues have similar appearances as nodules on CT images. There are more normal lung tissues in the central area, especially near the hilar region. It is sometimes challenging for doctors to distinguish lung nodules from pulmonary vessels, bones, and other structures 11. This will cause difficulties in interpretation, resulting in a decrease in accuracy. When lung tissues are regarded as lung nodules, they cause an increase in FP. Conversely, if the existing lung nodules are regarded as lung tissues, the sensitivity will be reduced. The approach for differentiating between the tissues and nodules is therefore crucial to reduce FP in an automatic lung nodule detection scheme 1. In our AI assistant programs, the FP rates were greatly decreased.

The characteristics of lung nodules are also a factor for detecting lung nodules on CT scans. Previously, Li et al. revealed that lung cancers missed on CT screenings are very subtle and appear as small faint nodules 14. Del Ciello et al. also showed that blurred and unclear margins are also factors of missed lung nodules 11. Benzakoun et al. also suggested that the ground-glass component may hinder software detection of attenuation differences with the surrounding parenchyma 15. They reported that partially solid nodules with a sensitivity of 72% were much better than pure ground-glass nodules with a sensitivity of 28% 15.

Compared with previous studies, our study showed a balance between sensitivity and FP. Although many previous studies have shown high identification sensitivity, their FP rates are also quite high. Cui et al. built on a 50-layer deep neural network and trained a large multi-center database; its deep learning algorithm showed a sensitivity of 91.0% but had 2FPs/case 10. The computer-aided detection algorithms proposed by Ali et al. showed an overall accuracy of 64.4% (sensitivity 58.9%, specificity 55.3%, PPV 54.2%, and NPV 60.0%) 16. Cao et al. produced a sensitivity of 90% and FP 1/scan on their 3-Dimentional convolutional neural network (3D-CNN) 17. Dou et al. also used 3D CNNs and showed a sensitivity of 90.7% and 4 FPs/case 18 which was similar to that of Setio et al., who used multiview convolutional networks to obtain a sensitivity of 90.1% and 4 FPs/case 19. These results showed high sensitivity, but their FP rates were also quite high at approximately 2-4 FPs/scan 10, 16, 17, 19.

The balance between the sensitivity and FP is important. Most previous models showed high sensitivity and high FP in CT screening of lung nodules using computer-aided detection. Modern technologies allow doctors to detect focal lung lesions more efficiently. However, FP is a critical problem in lung nodule detection because FP results in unnecessary follow-up tests and expenditures 16. It also leads to increased patient suffering and even unnecessary invasive procedures to confirm the diagnosis. This results in an increased risk of procedures. In addition, frequent and regular follow-up of chest CT scans may also lead to radiation-induced cancer 20. Previous studies also showed that computer-aided detection software shows high FP, which represents a major limitation in the wider use of the system 11. Therefore, most computer-aided detection does not show evidence of benefits in the real world 20. FP reduction is a critical issue in AI lung nodule detection. In our current study, our AI-assisted model can greatly reduce the FP rate and improve the performance of doctors.

## Limitations of the study

Our AI-assisted model improved the accuracy of reading CT scans and the work efficiency of doctors. However, this study has some limitations. First, this research focused on the detection of lung nodules, but did not focus on the differentiation of benign and malignant nodules. We did not analyze the performance of the lung cancer using the current model. Therefore, such an assistant system requires further research to confirm the diagnosis of lung cancers. However, through early detection of small lung nodules, we believe that early detection of lung cancer is still helpful. Second, the lack of a gold standard is a common problem in the AI detection of lung nodules 10. Biopsy of lung nodules can confirm the correctness, but this is not feasible in most cases. In this study, we used the consensus of most experts as the reference standard. This approach was similar to that of previous studies on AI detection of lung nodules 10. Third, the incidence of lung nodules varies with different characteristics of the study population, such as race, age, and smoking status. Therefore, the accuracy of AI differs for different populations 10, 20. Our current system still needs to be used in other ethnic groups.

## Conclusions

Missed lung cancer has potentially serious medicolegal implications for doctors. The reasons for misdiagnosis on CT scans are related to specific characteristics of the undetected lesion, such as small size, ground-glass appearance, and central location. Manual detection with a large number of CT scans is a great burden, requires attention, is very time-consuming for doctors, and is prone to errors. In our study, AI assistant programs decreased the incidence of misinterpretation of lung nodules in the error-prone characteristics of lung nodules.

## Figures and Tables

**Table 1:** Demographic characteristics of the patients

Characteristics		
Age (yrs)		62.6±11.0
BH (cm)		159.8±8.8
BW(Kg)		61.1±13.6
Gender	Male	27 (45%)
	Female	33 (55%)
Smoking	Non-smoking	44 (73.3%)
	Current smoker	4 (6.7%)
	Ex-smoker	12 (20.0%)

Abbreviations: BH, body height; BW, body weight.

**Table 2:** The false positive and sensitivity of AI detection in different location, size and texture

		**Location**						**Location**					
		Left		Central		Right		Upper		Middle		Lower	
		FP/scan	Sensitivity	FP/scan	Sensitivity	FP/scan	Sensitivity	FP/scan	Sensitivity	FP/scan	Sensitivity	FP/scan	Sensitivity
without AI	mean	0.125	70.9%	0.334	55.6%	0.176	74.5%	0.142	69.8%	0.300	58.5%	0.184	44.5%
	lower 95% CI	0.006	51.1%	0.316	40.7%	0.153	65.2%	0.024	60.5%	0.206	40.1%	0.000	42.7%
	upper 95% CI	0.243	90.6%	0.352	70.3%	0.197	83.6%	0.258	78.9%	0.393	76.8%	0.419	46.3%
with AI	mean	0.011	71.6%	0.094	68.9%	0.017	69.7%	0.055	77.6%	0.033	66.1%	0.017	45.4%
	lower 95% CI	0.000	51.2%	0.043	50.1%	0.000	52.4%	0.004	60.8%	0.003	43.9%	0.000	31.0%
	upper 95% CI	0.049	92.1%	0.145	87.6%	0.074	87.0%	0.106	94.2%	0.066	88.2%	0.049	59.7%
		**Size**						**Texture**					
		Small		Middle		Large		GGO		Partial solid		solid	
		FP/scan	Sensitivity	FP/scan	Sensitivity	FP/scan	Sensitivity	FP/scan	Sensitivity	FP/scan	Sensitivity	FP/scan	Sensitivity
without AI	mean	0	3.2%	0.192	69.4%	0.442	80.2%	0.409	65.1%	0.100	74.6%	0.125	57.9%
	lower 95% CI	0	3.2%	0.000	40.3%	0.041	77.4%	0.101	64.7%	0.000	61.3%	0.102	39.5%
	upper 95% CI	0	3.2%	0.545	98.3%	0.841	82.8%	0.715	65.5%	0.334	87.8%	0.147	76.2%
with AI	mean	0.077	76.3%	0.011	80.3%	0.033	59.3%	0.039	61.5%	0.056	74.5%	0.078	74.7%
	lower 95% CI	0.000	57.5%	0.000	67.3%	0.000	35.3%	0.000	43.6%	0.000	63.6%	0.000	51.6%
	upper 95% CI	0.177	94.9%	0.030	93.3%	0.066	83.3%	0.108	79.3%	0.219	85.2%	0.173	97.7%
